# Effect of Perinatal Dioxin Exposure Originating from Agent Orange on Gaze Behavior in 3-Year-Old Children Living in the Most Dioxin-Contaminated Areas in Vietnam

**DOI:** 10.3390/toxics10040150

**Published:** 2022-03-22

**Authors:** Thao Ngoc Pham, Muneko Nishijo, Tai Pham-The, Nghi Ngoc Tran, Hoa Thi Vu, Anh Hai Tran, Tien Viet Tran, Yoshikazu Nishino, Hisao Nishijo

**Affiliations:** 1 Department of Epidemiology and Public Health, Kanazawa Medical University, Ishikawa 920-0293, Japan; phamngocthaovmmu@gmail.com (T.N.P.); vuhoa5593hvqy@gmail.com (H.T.V.); ynishino@kanazawa-med.ac.jp (Y.N.); 2 Department of Functional Diagnosis, 103 Military Hospital, Vietnam Military Medical University, Hanoi 12108, Vietnam; tientv@vmmu.edu.vn; 3 Biomedical and Pharmaceutical Research Centre, Vietnamese Military Medical University, Hanoi 12108, Vietnam; taithuy@kanazawa-med.ac.jp (T.P.-T.); anhhtr@yahoo.com (A.H.T.); 4 Ministry of Health, Vietnamese Government, Hanoi 10060, Vietnam; nghi_tranngoc@yahoo.com; 5 Faculty of Medicine, University of Toyama, Toyama 930-0194, Japan; nishijo@med.u-toyama.ac.jp

**Keywords:** dioxin, gaze behavior, autistic trait, social communication, Vietnam

## Abstract

We investigated the effect of perinatal dioxin exposure indicated by dioxins in breast milk on children’s gaze behavior. We studied 142 children aged 3 years from the 2012 Bien Hoa birth cohort in a hotspot of dioxin contamination in Vietnam. Children’s faces were viewed using the eye-tracking method. Associations between gaze behavior of faces and neurodevelopmental indices and head circumference were analyzed to determine whether poor gaze behavior indicates increased autistic traits in these children. The gaze fixation duration on facial areas when viewing 10 still images of children was calculated as the gaze behavior index. Autistic behavior was assessed using the Autism Spectrum Rating Scale, and language development was evaluated by the Bayley Scales of Infant and Toddler Development, Ver. 3. The face fixation duration (%) significantly decreased as 2,3,7,8-tetrachlorodibenzo-p-dioxin (TCDD) concentrations increased in a dose–effect manner in girls, which suggested atypical gaze behavior for watching human faces. Furthermore, these girls with atypical gaze behavior showed lower social communication scores and smaller head sizes, suggesting increased autistic traits in girls. In conclusion, our findings show sex-specific effects (girls > boys) of perinatal TCDD exposure on gaze behavior in young children.

## 1. Introduction

During Operation Ranch Hand by the US military in Vietnam from 1961 to 1971, a large quantity of Agent Orange containing 2,3,7,8-tetrachlorodibenzo-p-dioxin (TCDD), which is the most toxic congener of dioxin, was stored and spilled during mixing and loading at several US airbases. This spillage caused considerable dioxin contamination in the environment and to people living at the airbase. This dioxin contamination of Bien Hoa airbase is the largest scale of contamination of an airbase, particularly from TCDD originating from Agent Orange. In 2012, we recruited 210 pairs of mothers and children living in 10 communities close to Bien Hoa airbase (Bien Hoa birth cohort 2012). We reported that mean TEQ-PCDD/Fs levels were 10.5 pg-TEQ/g lipid for primipara and 8.9 pg-TEQ/g lipid for multipara and significantly higher than those in unsprayed areas (3.5 g-TEQ/g lipid for primipara and 3.0 pg-TEQ/g lipid for multipara) [1] and 5.9 pg-TEQ/g of critical maternal level suggested by EFSA Panel on Contaminants in the Food Chain (CONTAM) in 2018 [2]. Moreover, mean TCDD concentrations in maternal breast milk were 2.6 pg/g lipid and approximately 4–5 times higher in primiparous mothers, and 2.2 pg/g lipid and 7–8 times higher in multiparous mothers compared with those in unsprayed areas (0.57 pg/g lipid for primipara and 0.31 pg/g lipid for multipara) [1].

TCDD is the most typical and powerful endocrine rupture chemical (EDC), whose toxicity is induced mainly via activation of the aryl hydrocarbon receptor (AhR) binding to the xenobiotic response element of the target gene [3]. AhR activation by TCDD during the perinatal period was reported to involve in the pathogenesis in developmental disorders associated with alteration of pituitary hormones including growth hormone and gonadotropins and steroid hormones whose synthesis is regulated by pituitary hormones in animals [4,5,6,7,8] and humans [9,10,11,12,13].

Interestingly, a high level of androgen exposure during the fetal period is suggested to increase the risk for autism spectrum disorder (ASD) [14,15,16,17], which is one of the neurodevelopmental disorders suspected to be prevalent with increasing prenatal EDC exposure [18].

To investigate the association between child neurodevelopment and dioxin exposure, particularly TCDD, we followed up with children from the Bien Hoa birth cohort 2012 and additionally collected 78 mother–child pairs in Bien Hoa in 2015 (Bien Hoa birth cohort 2015). We then found that children who were perinatally exposed to TCDD at high concentrations (5.5 pg/g lipid of dioxins in breast milk) showed significantly lower expressive language than that in boys at 2 years of age [19]. We also examined neonatal electroencephalograms (EEGs) in children from the Bien Hoa birth cohort 2015 and found that perinatal TCDD exposure altered EEG power values. This alteration led to poor gaze behavior at 2 years of age as indicated by a decreased fixation duration on the face of a child in a video presented on a screen. These results suggest that prenatal TCDD exposure affects fetal brain development and may lead to a poor communication ability among them [20].

Since a reduced fixation duration on faces has frequently been observed in children who have difficulties in social interactions or poor neurodevelopment, such as children with autism [21,22,23], we examined gaze behavior for viewing static human facial images in children from the Bien Hoa birth cohort 2012. We reported that perinatal exposure to high TCDD exposure may decrease the percentage of the face fixation duration in girls [24]. At that time, however, the associations between gaze behavior and their neurodevelopmental indices, including autistic behavior as indicated by Autism Spectrum Rating Scales (ASRS) scores, were not investigated at that time. We also did not analyze the associations between gaze behavior and head circumference, which are larger in children with autism [25,26,27] or smaller in girls with autism [28] compared with those in normally developed children or girls.

Therefore, in the present study, we first confirmed the effect of perinatal dioxin exposure, particularly TCDD exposure, on gaze behavior in our previous report [24]. Then, we investigated the associations between gaze behavior and indicators of autistic traits, including ASRS scores and head circumference, in 3-year-old children living in the most dioxin-contaminated areas in Vietnam.

## 2. Materials and Methods

### 2.1. Study Areas and Subjects

#### 2.1.1. Study Areas

Bien Hoa airbase is one of the largest former US airbases examined dioxins and heavy dioxin contamination with high TCDD contribution to total dioxins (>80%) was reported in soil and sediment of canals and ponds in the airbases with 61,400 (pg/g dry wet) for the highest concentration of TCDD in soil samples [29]. Shecter et al. (2001) reported higher TCDD in milk samples collected from residents nearby Bien Hoa airbase with 133–1832 (pg/g lipid) in 1970–1973, 2.1–11 (pg-g lipid) in 1985–1988, and 2.0–164 (pg/g lipid) in 1999 compared with the unsprayed area (<2 pg/g lipid) [30].

#### 2.1.2. Study Subjects

In 2012, 224 mother–child pairs living in 10 communities close to Bien Hoa airbase were recruited for a baseline study between September and December (Bien Hoa birth cohort 2012). The mothers delivered their newborns at Dong Nai Prefectural Hospital in Bien Hoa City, Vietnam. The following criteria were used for inclusion in the study: (i) the mothers resided in the target area at least during pregnancy; (ii) the newborns were born full-term, and (iii) there were no complications during birth [1]. A summary of follow-up surveys of the present cohort was shown in Table 1.

A follow-up study at 3 years of age was carried out between November and December 2015. A total of 193 (86.2%) children participated in a survey to examine gaze behavior and their neurodevelopment. However, gaze data from 40 children were missing. Eight children (5 boys and 3 girls) could not attend the gaze test because the electricity was cut-off on the examination day at one of the local survey sites in the community. Eighteen children (5 boys and 13 girls) refused to participate in the gaze test or did not have a successful calibration of the eye-tracking system before the gaze test. Fourteen children (7 boys and 7 girls) showed a lack of attention because the total duration of fixation on the screen was <1.13 s (10th percentile), and they were excluded from the data set. Moreover, 11 children were excluded because of missing data as follows. Seven children did not have dioxin concentrations measured in breast milk because of an insufficient volume of breast milk samples. Two children had missing information regarding the smoking status of family members. Two children were missing ASRS scores because family members who accompanied the children did not know their behavior well. There were no significant differences regarding characteristics or breast milk dioxin concentrations between participants and non-participants or included and excluded subjects. Therefore, the final number of children included for data analysis was 142 (80 boys and 62 girls).

Data on the characteristics of the parents (age, education, family income, primipara/multipara, smoking, and alcohol drinking) and the children (gestational weeks and sex) were collected and are shown in Table 1. The mean maternal age and duration of education with standard deviation (SD) in total participants to the 3-year-old survey in both sexes were 28.5 (4.8) and 11.3 (3.2) years, respectively. The number of primiparous mothers was 55 (38.7%). No mother was a smoker, but 66.9% of mothers stayed with family members who smoked. The proportion of mothers who consumed a product of alcohol during pregnancy was 4.9%, but this was only occasionally (<300 mL of beer each time). The mean gestation was 39.0 weeks and the rate of boys was 56.3%. For these maternal characteristics and family monthly income, there was no significant difference between total cohort pairs and participant pairs (statistic test results were not shown).

Mean values and Z-scores of the children’s body size (weight, length/height, body mass index, and head circumference at birth and at 3 years) are shown in Table 1. Body size values were adjusted for age and sex to be standardized Z-scores by following the World Health Organization (WHO) standards (www.who.int/childgrowth/, (accessed on 11 March 2022)). No difference in Z-scores of body size indices at birth was found between total cohort pairs and participant pairs (statistic test results were not shown). Additionally, there was no difference in Z-scores of body sizes at birth and at 3 years of age between boys and girls, except for the head circumference at birth, which showed borderline significance (*p* = 0.053).

Written informed consent was obtained from all of the mothers according to a process that had been reviewed and approved by the Health Department of Dong Nai Province and the Vietnam Military Medical University. The institutional ethics board for epidemiological studies at Kanazawa Medical University approved the study design (No. E-187).

### 2.2. Dioxin Measurements

When the infants were approximately 1 month old, nurses from a community health station visited the mother’s house and collected approximately 20 mL of maternal breast milk. These samples were frozen and transported to Japan inside dry ice by airplane. Approximately 10 mL of breast milk for each sample was used to quantify the levels of 7 congeners of polychlorinated dibenzo-p-dioxins (PCDDs) and 10 congeners of polychlorinated dibenzofurans (PCDFs) in the High Technology Center at Kanazawa Medical University in Uchinada, Japan [31]. After using the EYELA freeze-dryer (FDU-1200; Tokyo-rika Inc., Tokyo, Japan) to freeze and dehydrate breast milk samples, the fat content (g) was extracted using the ASE-200 accelerated solvent extractor (Dionex Co., Sunnyvale, CA, USA). We then added 13C-labeled 2,3,7,8-substituted PCDD/Fs (DF-LCS-A40; Wellington Inc., Guelph, ON, Canada) as an internal standard. A series of procedures, including alkali digestion, hexane extraction, and chromatography on a multilayered silica gel column, were performed to purify samples. A single-layered column of activated carbon was used to separate and collect PCDD/PCDF fractions. Concentrations of 17 PCDD and PCDF congeners were quantified using a gas chromatograph (HP-6980; Hewlett-Packard, Palo Alto, CA, USA) equipped with a high-resolution mass spectrometer (high-resolution-gas chromatography/mass spectrometry, MStation-JMS700; JEOL, Tokyo, Japan) operating in the selected ion-monitoring mode. Toxic equivalent (TEQ)-PCDD/Fs of 7 PCDD and 10 PCDF congeners were calculated as the sum of all values, which were obtained by multiplying each congener concentration by its toxic equivalent factor from WHO 2005-TEF [32]. Congeners that showed a concentration below the detection limit were set to half of the detection limit. The method for analysis has been described in more detail in previous studies [1,33].

TCDD was detected in all samples. Geometrical means and geometrical standard deviations of TCDD and TEQ-PCDD/Fs in maternal breast milk are shown in Table 2. The geometrical mean of TCDD and TEQ-PCDD/F concentrations was 2.5 pg/g lipid and 9.6 pg-TEQ/g lipid, respectively.

### 2.3. Gaze Behavior Examination

The Tobii X2-60 Compact eye tracker running at 60 Hz (Tobii Technology, Stockholm, Sweden) was used to examine gaze behavior in children aged 3 years. The position of both eyes was recorded by an infrared camera below the screen of a computer. Children were comfortably seated on the lap of their mother or caregiver with a distance from the child’s eyes to the screen of approximately 60 cm and with free viewing. At first, 10 static images showing animals and some familiar objects, such as a cake, bicycle, and a pair of scissors, were shown for children as a practice phase. This viewing was performed to attract attention from children toward the screen. Ten static images of the human face were then shown to children as gaze stimuli, with 3 s for each picture. Before recording, children had to be successfully calibrated by the eye-tracking system with five red points, which appeared at the center and four corners of the screen.

**Figure 1:**
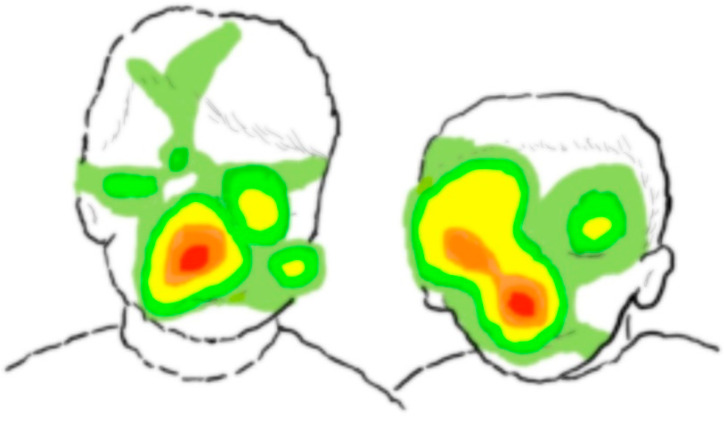
Density of the fixation duration when children watched a picture of two children.

We used Tobii Studio version 3.3.1 (Tobii Technology, Stockholm, Sweden) to extract children’s data and analyze their fixation duration. Before extracting data, the default I-VT filter was applied with the velocity threshold and minimum fixation duration set at 30 degrees/s and 100 ms, respectively, to determine fixation. Figure 1 shows the fixation density of the child when they viewed a picture. Whole picture and face areas were manually set to define areas of interest in each picture. The total fixation duration on the whole picture and face areas in each picture (by second) were extracted by Tobii software. The total fixation duration on pictures and face areas was calculated by adding up these values for 10 pictures. The percentage of the total fixation duration on faces was defined as the ratio of the total fixation duration on faces in all pictures divided by the total fixation duration (s) in all pictures, and then the ratio was multiplied by 100 to obtain the percentage of face fixation. This method was described in more detail in our previous studies [20,24,34].

### 2.4. Neurodevelopmental Assessment

General neurodevelopment was examined using the Bayley III (NCS Pearson, Inc., Bloomington, MN, USA) across five domains of cognition, expressive and receptive language, and fine and gross motor skills. Two examiners performed this test in our previous study [19], who were trained well and blinded to dioxin exposure levels of the children who were administered the tests. At this time, cognition and language ability were assessed by one examiner, and another examiner assessed motor development in all children.

Full-length parent rating forms (2–5 years) of the ASRS (MSH, North Tonawanda, NY, USA) with 70 questions that assess child behavior associated with autism spectrum disorder (ASD) were used to interview mothers or caregivers of children in this study. Before the survey, a trial examination was conducted on a group of 15 Vietnamese children to ensure the feasibility and appropriateness of the ASRS for the Vietnamese population [35]. The total score, the Diagnostic and Statistical Manual of Mental Disorders, 4th edition, Text Revision (DSM-IV-TR; DSM) score, the social communication score, and the Unusual Behavior score were calculated after the interview by following the ASRS guidebook (ASRS, Technical Manual). The total score and DSM scores were >60, which is suspected to have autistic traits. One medical doctor was trained well by a specialist and interviewed all subjects in this study. The detailed method was described in a previous study [35].

### 2.5. Statistical Analysis

The IBM SPSS (ver. 21) software package for Windows (IBM Corp., Armonk, NY, USA) was used for statistical analysis. Concentrations of 17 PCDD/F congeners and TEQ-PCDD/Fs in breast milk were logarithmically transformed (base 10) to improve normality. A general linear model was used to compare the face fixation duration (%) between the high and low exposure groups after adjusting covariates. The cut-off value for high and low TCDD concentrations was 3.5 pg/g lipid. This value was based on the geometrical mean and the geometrical SD of dioxin concentrations in breast milk samples from unsprayed areas reported in our previous study using the following equation: geometrical mean × geometrical SD^3^ [36]. For the cut-off value of TEQ-PCDD/Fs, the 75th percentile value of TEQ-PCDD/Fs in the subjects was used because of comparatively lower concentrations than those in another dioxin hotspot around Da Nang airbase [33,36]. The following factors were included as covariates: maternal age and education (years), parity (primipara/multipara), alcohol consumption during pregnancy (yes/no), family income, family member smoking (yes/no), and gestational weeks. To analyze the dose–effect relationship between TCDD concentrations and the face fixation duration (%), subjects were divided into three groups (low, middle, and high) with cut-off values of 3.5 and 6.3 pg/g lipid. At this time, the 90th percentile value of TCDD in these subjects was used for the higher cut-off value. Differences among the groups were analyzed using the general linear model.

For analyses of associations between the face fixation duration (%) and neurodevelopmental indices or body size, including the head circumference, we used the linear regression model after adjusting for the same covariates as those used in general linear model analysis. For all analyses, significance was considered *p* < 0.05.

## 3. Results

### 3.1. Effects of Perinatal TCDD and TEQ-PCDD/F Exposure on Gaze Behavior

We compared the adjusted mean face fixation duration (%) between the high and low exposure groups according to TCDD or TEQ-PCDD/F concentrations in boys and girls (Table 3). No significant difference in the face fixation duration (%) was found between the high and low TCDD exposure groups in boys. However, in girls, the adjusted mean face fixation duration (%) was significantly lower (*p* < 0.05) in the high TCDD group compared with that in the low TCDD group. However, no significant difference in the face fixation duration (%) was found between the high and low TEQ-PCDD/F exposure groups in both sexes (Table 3).

**Figure 2:**
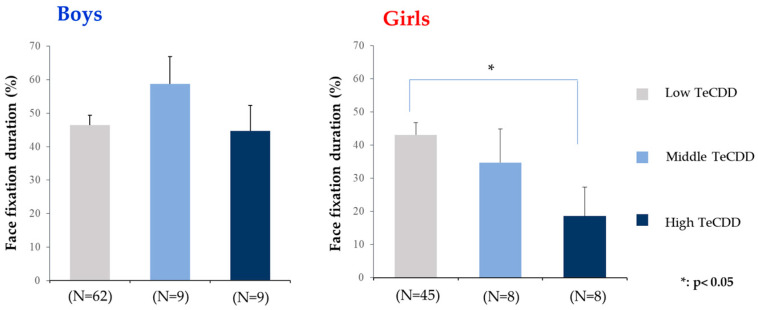
The dose–effect relationship between perinatal TCDD exposure and gaze performance: comparison of the adjusted face fixation duration (%) among the low (<3.5), middle (3.5–6.3), and high (≥6.3 pg/g lipid) TCDD groups in boys and girls. Note: TCDD: 2,3,7,8-tetrachlorodibenzo-p-dioxin; N: number of subjects.

To clarify the dose–effect relationship between perinatal TCDD exposure and gaze performance, the mean face fixation duration (%) was compared among the three TCDD exposure groups of low (<3.5), middle (3.5–6.3), and high (≥6.3) (pg/g lipid), after adjusting for covariates (Figure 2). In girls, the adjusted mean face fixation duration (%) significantly decreased (*p* < 0.05) as exposure levels of TCDD increased. However, in boys, no dose–response relationship was found between TCDD concentrations and the face fixation duration (%).

At this time, we did not analyze the dose–effect relationship with TEQ-PCDD/Fs exposure because the rates of high TEQ-PCDD/Fs (≥12.5 pg-TEQ/g lipid) were 10.3, 52.6, and 93.7 (%) for low, middle, and high TCDD groups, suggesting TCDD is a good exposure marker to show a wide range of dose in the present study.

### 3.2. Gaze Behavior and Neurodevelopment as Indicated by Bayley III and ASRS Scores

To investigate the relationships between gaze behavior and neurodevelopment, including autistic traits, the associations between the face fixation duration (%) and Bayley III and ASRS scores were analyzed using the regression linear model. An adjustment was made for the same covariates that were used in the general linear model analysis (Table 4). There was no significant association between the face fixation duration (%) and Bayley III scores in either sex. However, in girls, the face fixation duration (%) increased with an increase in composite and receptive language scores (β = 0.293 and β = 0.245, respectively; *p* = 0.058 and *p* = 0.064, respectively).

No significant association of ASRS scores was observed in boys. In girls, the face fixation duration (%) was inversely and significantly associated (*p* < 0.05) with social communication scores, where a lower face fixation duration (%) was associated with higher social communication scores (poor social communication skills).

We also compared the adjusted mean Bayley III and ASRS scores between the high and low TCDD groups using the general linear model. However, there were no significant differences in any scale scores of the ASRS or Bayley III between the two TCDD exposure groups in girls (data not shown).

### 3.3. Gaze Behavior and Head Circumference at Birth and 3 Years of Age

The associations between the face fixation duration (%) and all body size indices (Z-score), which comprised weight, length/height, head circumference, and body mass index, at birth and at 3 years of age were analyzed after adjusting for covariates in each sex (Table 5). In boys, there were no significant associations between the face fixation duration (%) and any body size indices at birth and at 3 years of age. However, the face fixation duration (%) was significantly and inversely associated (*p* < 0.05) with height at 3 years of age in girls. The face fixation duration (%) appeared to increase as the head circumference increased (β = 0.230), but this association was not significant (*p* = 0.125).

Because the head circumference was correlated with height, a regression analysis between the face fixation duration (%) and head circumference at 3 years of age was performed again after adjusting for covariates, including height at 3 years of age, in both sexes (Table 5). The association between the face fixation duration (%) and head circumference at 3 years of age was significantly increased after adjusting for height (β = 0.281, *p* < 0.05), which suggested that children with a lower gaze fixation (%) on the face had a smaller head size.

## 4. Discussion

### 4.1. Effect of Perinatal Dioxin Exposure on Gaze Behavior in 3-Year-Old Children

In girls aged 3 years from the Bien Hoa cohort 2012, the gaze fixation duration on faces of static pictures decreased as perinatal TCDD exposure increased in a dose–effect manner. This finding suggested that atypical gaze behavior was associated with TCDD exposure in girls. However, no association was observed between dioxin congeners other than TCDD and gaze behavior in either sex, which indicated that TCDD is the only congener that affects the gaze behavior of children.

Gaze behavior has been frequently examined in children with autism or poor general neurodevelopment, and they have atypical gaze behavior with fewer fixations on the whole face compared with children without these conditions [21,22,23]. However, previous studies on the effects of organochlorine compounds on gaze behavior are limited, except for one report on polychlorinated biphenyl congeners and gaze behavior in Japanese infants [37]. This Japanese study reported that infants who were prenatally exposed to high concentrations of polychlorinated biphenyl #118 preferred to fix their gaze on inverted point-light displays of a walking human figure rather than reduce attention to upright biological motion.

In the Bien Hoa birth cohort 2015, we found that perinatal TCDD altered neonatal EEG power values, which were associated with a reduced face fixation duration (%) in children aged 2 years when viewing dynamic social stimuli (a movie of a young girl playing in front of her mother taking a video of her) [20]. However, different gaze stimuli in this previous study were used compared with those used in the present study. Moreover, we previously reported that perinatal TCDD exposure may decrease face fixation duration in 3-year-old girls from the Bien Hoa birth cohort 2012 based on the same dataset as that used in the present study [24]. In the present study, we found a clearer dose–response relationship between TCDD exposure and the face fixation duration in girls after excluding more children with inattention compared with that in the previous analysis.

In the present analysis, we analyzed the associations between gaze behavior and concentrations of PCDD congeners other than TCDD, but no significant difference was observed between the high and low exposure groups (data not shown). We also found that autistic traits increased with an increase in TCDD concentrations, but not TEQ-PCDD/Fs, in 3-year-old children in our previous study in children from the Da Nang birth cohort [35]. However, Nowack et al. (2015) reported an effect of prenatal exposure to TEQ-PCDD/Fs on autistic traits in childhood using the Social Responsiveness Scale in children from the Duisburg birth cohort in Germany [38]. Therefore, we need to follow up with these children to determine whether TCDD is a specific congener that increases autistic behavior, including gaze behavior, on faces in the future.

### 4.2. Gaze Behavior and Child Neurodevelopment including Autistic Traits

In the present study, in girls, the face fixation duration (%) significantly decreased as social communication scores of the ASRS increased. This finding suggested poor social communication skills, which are often observed in children with autism, although no direct association was found between TCDD exposure and ASRS scores. The face fixation duration (%) also tended to decrease as Bayley III language scores decreased in girls, which suggested that a decreased gaze fixation on faces indicated a poor communication ability. These results suggest that a decreased face fixation duration may be associated with increased autistic traits in children from the Bien Hoa birth cohort 2012.

Gaze behavior toward faces, particularly the eyes and mouth regions, has been investigated using eye-tracking of static pictures or video clips in individuals with autism, because of social communication deficit which is one of their characteristic symptoms [39]. Previous studies showed decreased gaze behavior in the faces indicated by shorter fixation duration on faces in children [21,23] and adults [22] with ASD compared with normally developed children and adults. Particularly in the eye regions, decreased gaze behavior was found in adults with ASD [22] but decreased gaze behavior was observed in the mouth regions in children with ASD [23].

We also analyzed gaze behavior in the eyes and mouth in the present study but found no significant difference in gaze behavior in the eyes or mouth regions between the high and low TCDD groups (data not shown). Nakano et al. (2010) used video clips with sound involving human characters who were talking to the audience or each other [23]. This type of dynamic stimulus may be more attractive for children and induce different gaze behavior from that for static pictures because of viewing a talking mouth and face and hearing voices. In the future, we will analyze the fixation duration on the face, eyes, and mouth regions after separating talking scenes from silent scenes, and clarify the atypical gaze pattern associated with dioxin exposure in Vietnamese children.

The risk of ASD in childhood is suspected to be increased by high androgen exposure during the fetal period [14,15,16,17]. It was also reported that aromatase expression and estrogen or estrogen receptor expression were associated with the development of ASD [40,41]. On the other hand, TCDD is the most powerful EDC which may alter the uterine endocrinal environment, particularly pituitary hormones including growth hormone and gonadotropins, and influence fetal development [4,5,6,7,8]. Taken together, perinatal TCDD exposure may influence the fetal neuroendocrine environment which contributes to pathology for ASD, leading to increased autistic traits in children exposed to TCDD in early life.

### 4.3. Gaze Behavior and Head Circumference

In the present study, the face fixation duration (%) decreased with an increase in height at 3 years of age in girls. This finding suggested that girls who had a shorter gaze fixation on faces were taller. In contrast, the face fixation duration (%) significantly decreased with a decrease in the head circumference at 3 years of age after adjusting for confounding factors including height in girls. This finding suggested that girls with a shorter fixed gaze on faces had a smaller head size relative to their height.

An increased rate of macrocephaly indicating a large brain volume has been suggested in children with autism in previous studies [25,26,27]. Lainhart et al. (2006) reported that the mean standardized head size was significantly increased relative to height in individuals with autism. They also investigated the distribution of height, which is correlated with the head circumference in autism, and showed no difference in height between individuals with and those without autism [27].

A recent meta-analysis of 12 studies included autistic and normally developed children [28]. This analysis showed that in girls with autism, the mean head circumference was smaller than that in normally developed girls at 12–17 months of age. Additionally, an extreme head circumference above and below 1.5 standard deviations was reported when autistic girls reached 36–59 months of age. However, there was no significant difference in the mean head circumference and extreme head circumference at the same age ranges in boys with autism. These results suggest that the head size may be affected by age and sex in autism and that the rate of a smaller head circumference may be more frequent at 3 years of age in girls. Therefore, 3-year-old girls who were perinatally exposed to high TCDD concentrations in the present study may have had a smaller head associated with increased autistic traits. This possibility suggests that changes in brain development are associated with TCDD exposure during the perinatal period.

In the present study, a shorter gaze fixation on faces was more likely in taller girls. Almost no evidence was found between gaze behavior, and autistic traits and height in previous literature, except for a study on children with high-functioning autism and Asperger disorder [42]. This previous study reported a high growth rate of length/height during the first 3 years of life in these children. In the present study, no effect of dioxin exposure on height was found in children of either sex (data not shown). Therefore, we need to follow up this birth cohort in the future to clarify the relationship between gaze behavior/autistic traits and body size parameters, including head circumference and height, in each sex.

### 4.4. Sex Differences in Dioxin Effects on Neurodevelopment and Behavior in Children

In a neurodevelopmental study at 2 years of age in children from the Bien Hoa birth cohorts 2012 and 2015 including the present subjects, a significantly lower expressive language score was associated with perinatal TCDD exposure only in boys [2]. Similarly, in follow-up studies in children from the Da Nang cohort, a significant decrease in motor and expressive language scores associated with perinatal TCDD and TEQ-PCDD/F exposure in the first 3 years of life [43] and decreased scores of cognitive ability and motor coordination skills associated with increased TEQ-PCDD/Fs at 5 years of age [44] were found only in boys. Poor learning ability was found only in 8-year-old boys exposed to high PCDD congener concentrations, including TCDD, during the perinatal period [45].

However, another study of children in Da Nang showed that increased ADHD symptoms, particularly impulsivity and hyperactivity, were associated with TCDD exposure only in girls [46]. In a previous study to examine feminine visual preference using eye-tracking of pictures of boy- or girl-oriented objects, we reported that visual interest in girl-oriented pictures was associated with high TCDD exposure in girls, and associated with PCDD congeners other than TCDD in boys [34]. Moreover, Vu et al. (2021) reported that mirror neuron activity indicating a reduction in EEG power by observation of hand movements, which plays an important role in social–emotional behavior, was altered with high perinatal TCDD exposure in girls at 9 years of age [47]. These results suggest that perinatal TCDD exposure might affect specific domains of neurodevelopment, such as social–emotional behavior, in older girls (early school age).

However, in most previous studies that investigated the association between dioxin exposure and neurodevelopment in children at similar ages in countries other than Vietnam, effects of dioxins were reported only in boys. In the Seveso Second Generation Health Study in Italy, Ames et al. (2019) reported that increased non-perseverative errors of the Wisconsin card sorting test were associated with perinatal TCDD exposure only in boys at 7–17 years of age [48]. In the Rotterdam cohort in The Netherlands, Vreugdenhil et al. (2002) reported that perinatal TEQ-PCDD/Fs exposure increased feminine behavior, which was more pronounced in boys than in girls at 8 years old [49]. In the Duisburg cohort in Germany, perinatal TEQ-PCDD/Fs exposure affected sex-typed behavior both in boys and girls aged 6–8 years [50].

These results indicating sex-patterned alteration of child behavior due to perinatal dioxin exposure might be related to characteristics of EDCs which alternately show estrogenic and androgenic effects in a sex-specific manner.

During the perinatal period, infants are suggested to be highly hormone-sensitive and influenced by TCDD exposure even at lower levels than the levels to induce systemic toxicity in adults based on animal studies [51,52] (bimodal effects of EDCs). In animal models, perinatal TCDD exposure at a low level reduced fetal luteinizing hormone (LH) levels by downregulation in the pituitary gene, leading to impaired sexual behavior in later life [53]. Since AhR has an important role in the homeostatic regulation of LH production in the pituitary gland [54], TCDD binding AhR is suggested to disrupt LH production and induce sexual immaturity in fetuses [55].

Taken together, we need to follow up our Vietnamese birth cohorts exposed to TCDD until adolescence when their hormonal environment will be drastically changed, and to investigate their pituitary hormonal levels and puberty arrival in the future.

### 4.5. Limitations

In this study, we investigated the effects of perinatal TCDD exposure on gaze behavior using static pictures as gaze stimuli. However, static pictures are likely to have a less natural form and motion than dynamic stimuli in a video clip and may induce different gaze behavior from that with watching faces in real life [56]. Therefore, further studies on gaze behavior in children using dynamic facial stimuli shown in a video clip are required.

Another limitation to the present study is its relatively small sample size because of the high rate (18.4%; 32 children/172 participants) of an unsuccessful gaze behavior examination. In particular, calibration with viewing five red points before recording was difficult for some children. A dynamic cartoon figure accompanied by sound for calibration instead of red points could be used to easily attract the children’s attention. Additionally, changing stimuli from static pictures to a movie of playing and talking children may improve the point of eye-tracking to make young participants pay more attention to face stimuli and achieve sufficient gaze behavior for analysis.

Eye tracking, which we used in the present study, is an objective tool for assessing gaze behavior to clarify children’s preferences and interests, which are not able to be obtained by a questionnaire survey. Further study using the eye-tracking method with a greater number of subjects, including unexposed control children, is necessary to clarify the association between dioxin exposure and atypical behavior in children from birth cohorts in Vietnam.

## 5. Conclusions

This study showed that 3-year-old girls living in a hot spot of dioxin contamination showed atypical gaze behavior when watching children’s faces in pictures that were proportional to perinatal TCDD exposure. These girls with atypical gaze behavior showed lower social communication scores and had a smaller head size, which can be found in girls with autism. We will follow up this birth cohort and investigate them again with an improved eye-tracking method to clarify the effects of perinatal dioxin exposure, particularly TCDD, on their behavior and hormonal levels in late childhood and adolescence in the future.

## Figures and Tables

**Table 1:** Summary of Bien Hoa birth cohort 2012.

Year	Age	Performance	N	Examinations
2012	at birth	Recruitment at the hospital	224	Body sizes
	1 month	Maternal breast milk collection	210	Body sizes
2014	2 years	Follow-up	174 *	Body sizes and neurodevelopment
2015	3 years	Follow-up	193	Body sizes and neurodevelopment
			153	Gaze behavior
			142	For present analysis

N: number of subjects. *: Pham TN reported the results of 227 children including these 174 children from the 2012 cohort and 53 children from the 2015 cohort.

**Table 2:** Characteristics of mothers and children and perinatal dioxin concentrations.

		All Cohort	Total (N = 142)	Boys (N = 80)	Girls (N = 62)
Characteristics	Units	Mean (SD), N (%)	Mean (SD), N (%)	Mean (SD), N (%)	Mean (SD), N (%)
*Mothers*					
Age	years	28.5 (4.6)	28.5 (4.8)	28.9 (4.9)	27.9 (4.6)
Education	years	11.3 (3.1)	11.3 (3.2)	11.4 (3.3)	11.2 (3.3)
Income (per a month)	million VND	9.8 (11.3)	10.2 (13.5)	9.6 (5.2)	11.0 (19.6)
Parity (% primipara)	N (%)	82 (37.6)	55 (38.7)	29 (36.3)	26 (41.9)
Alcohol drinking	N (%)	10 (4.7)	7 (4.9)	6 (7.5)	1 (1.6)
Family smoking	N (%)	140 (65.4)	95 (66.9)	50 (62.5)	45 (72.6)
*Children*					
Gender (rate of boys)	N (%)	116 (53.2)	80 (56.3)		
Gestational period	weeks	39.0 (1.2)	39.0 (1.3)	39.0 (1.3)	39.0 (1.2)
*At birth*					
Weight	g	3297 (411)	3272 (416)	3353 (425)	3168 (383)
	Z-score	0.01 (0.82)	−0.05 (0.82)	0.014 (0.83)	−0.13 (0.80)
Length	cm	49.9 (2.2)	49.8 (2.1)	50.1 (2.0)	49.5 (2.2)
	Z-score	0.18 (1.19)	−0.04 (2.48)	0.12 (1.04)	−0.25 (3.58)
Head circumference	cm	34.2 (3.4)	34.0 (3.8)	34.5 (3.2)	33.4 (1.8)
	Z-score	0.12 (2.10)	0.03 (2.18)	0.34 (2.54)	−0.38 (1.51)
BMI		13.3 (1.4)	13.2 (1.4)	13.3(1.4)	13.0 (1.3)
	Z-score	−0.09 (1.03)	−0.15 (1.04)	−0.05 (1.08)	−0.27 (0.99)
*At 3 years old*					
Age	month		37.5 (0.7)	37.5 (0.8)	37.6 (0.6)
Weight ^#^	kg		15.2 (2.7)	15.5 (2.8)	14.7 (2.6)
	Z-score		0.41 (1.35)	0.52 (1.47)	0.26 (1.17)
Height ^#^	cm		95.1 (3.5)	95.3 (3.5)	94.9 (3.5)
	Z-score		−0.32 (0.92)	−0.38 (0.94)	−0.23 (0.89)
Head circumference ^#^	cm		48.5 (1.5)	49.0 (1.4)	47.7 (1.3)
	Z-score		−0.48 (0.95)	−0.38 (0.98)	−0.60 (0.91)
BMI ^#^			16.7 (2.2)	16.9 (2.2)	16.2 (2.1)
	Z-score		0.88 (1.65)	1.07 (1.75)	0.63 (1.50)
*Breast milk*					
TCDD	GM (GSD)	2.3 (2.3)	2.4 (2.3)	2.4 (2.2)	2.4 (2.5)
TEQ-PCDD/Fs	GM (GSD)	9.1 (1.7)	9.4 (1.7)	9.5 (1.7)	9.3 (1.7)

N: number of subjects; SD: standard deviation; VND: Vietnam Dong per month; alcohol drinking: drinking habits during pregnancy; GM: geometrical mean; GSD: geometrical standard deviation; BMI: body mass index; Z-score: adjusted by age and sex with WHO reference; TCDD: 2,3,7,8-tetrachlorodibenzo-p-dioxin, TEQ-PCDD/Fs: toxic equivalency of polychlorinated dibenzo-p-dioxins and polychlorinated dibenzo furans; ^#^: missing for 1 boy and 1 girl.

**Table 3:** Comparison of the adjusted mean face fixation duration (%) between the high and low dioxin exposure groups.

	Low Exposure Group				High Exposure Group				
	N	Mean	adj. Mean	95% CI (Lower, Upper)	N	Mean	adj. Mean	95% CI (Lower, Upper)	*p*-Value
**Boys**									
TCDD	62	48.0	46.6	(40.8, 52.4)	18	46.6	51.3	(39.8, 62.8)	0.483
TEQ-PCDDs/Fs	60	45.7	45.6	(39.7, 51.5)	20	53.7	53.8	(43.2, 64.5)	0.194
**Girls**									
TCDD	45	43.1	43.4	(35.8, 50.9)	17	26.3	25.5	(12.7, 38.3)	0.025
TEQ-PCDDs/Fs	46	40.1	40.2	(32.5, 47.8)	16	33.8	33.6	(20.1, 47.0)	0.410

TCDD: 2,3,7,8-tetrachlorodibenzo-p-dioxin; TEQ: toxic equivalent; PCDD/Fs: polychlorinated dibenzo-p-dioxins and polychlorinated dibenzo furans; cut-off values: 3.5 (pg/g lipid) for TCDD, 12.5 (pg-TEQ/g lipid) for TEQ-PCDD/Fs; N: number of subjects; adj.Mean: adjusted mean, 95% CI: 95% confidence interval; covariates: age, education, parity, and drinking during pregnancy of mothers, family income, family members’ smoking habit, and gestational weeks, birth weight, and age (months) at the examination of children.

**Table 4:** Associations between the face fixation duration (%) and neurodevelopmental scale scores after adjusting for covariates.

	Boys				Girls			
	N	β	95% CI (Lower, Upper)	*p*-Value	N	β	95% CI (Lower, Upper)	*p*-Value
**Bayley III**								
Congnition	80	−0.081	(−0.381, 0.220)	0.594	59	0.137	(−0.140, 0.360)	0.380
Language (composite)	77	0.010	(−0.208, 0.226)	0.934	56	0.293	(−0.011, 0.653)	0.058
Receptive language	79	0.021	(−0.203, 0.242)	0.864	57	0.245	(−0.019, 0.616)	0.064
Expressive language	77	0.043	(−0.171, 0.247)	0.717	56	0.236	(−0.083, 0.599)	0.135
Motor (composite)	78	0.166	(−0.063, 0.350)	0.170	60	0.091	(−0.234, 0.448)	0.533
Fine Motor	80	0.113	(−0.110, 0.309)	0.348	61	−0.079	(−0.439, 0.258)	0.604
Gross Motor	78	0.159	(−0.069, 0.350)	0.184	60	0.195	(−0.111, 0.556)	0.186
**ASRS**								
Social communication	80	−0.132	(−0.327, 0.092)	0.267	62	−0.290	(−0.637, −0.019)	0.038
Unusual behavior	80	0.022	(−0.193, 0.233)	0.854	62	0.027	(−0.287, 0.347)	0.852
Total score	80	−0.066	(−0.263, 0.147)	0.574	62	−0.193	(−0.547, 0.101)	0.173
DSM-score	80	−0.031	(−0.234, 0.179)	0.792	62	−0.224	(−0.574, 0.060)	0.110

N: number of subjects; β: standardized beta; 95% CI: 95% confidence interval; Bayley III: the Bayley Scales of Infant and Toddler Development, Ver. 3; ASRS: autism spectrum rating scale; DSM: the DSM-IV-TR score; covariates: age, education, parity, and drinking during pregnancy of mothers, family income, family members’ smoking habit, and gestational weeks, birth weight, and age (months) at the examination of children.

**Table 5:** Associations between the face fixation duration (%) and standardized body size indices (Z-scores) after adjusting for covariates.

	Boys				Girls			
	N	β	95% CI (Lower, Upper)	*p*-Value	N	β	95% CI (Lower, Upper)	*p*-Value
At birth								
Weight	80	0.236	(−0.030, 0.473)	0.085	62	−0.115	(−0.415, 0.173)	0.412
Lenght	80	0.210	(−0.118, 1.074)	0.115	62	−0.185	(−0.334, 0.070)	0.195
BMI	80	0.097	(−0.139, 0.317)	0.438	62	0.113	(−0.194, 0.439)	0.440
Head circuference	80	0.075	(−0.129, 0.252)	0.522	61	0.051	(−0.366, 0.517)	0.733
At 3 years of age								
Weight	79	0.026	(−0.181, 0.227)	0.824	61	−0.110	(−0.495, 0.232)	0.472
Height	79	0.024	(−0.217, 0.262)	0.852	61	−0.292	(−0.612, −0.013)	0.041
BMI	79	0.029	(−0.179, 0.232)	0.799	61	0.037	(−0.316, 0.400)	0.814
Head circuference	79	0.027	(−0.192, 0.242)	0.817	61	0.230	(−0.071, 0.568)	0.125
Head circumference *	78	0.038	(−0.197, 0.267)	0.765	61	0.281	(0.004, 0.605)	0.047

N: number of subjects; β: standardized beta; 95% CI: 95% confidence interval; BMI: body mass index; covariates at birth: age, education, parity, and drinking during pregnancy of mothers, family income, family me mbers’ smoking habit, and gestational weeks at birth; covariates at 3 years of age: age, education, parity, and drinking during pregnancy of mothers, family income, family members’ smoking habit, and gestational weeks, birth weight, and age (months) at the examination of children; *: height at 3 years of age was added to covariates above.

## Data Availability

The data presented in this study are available on request to the corresponding author. The data are not publicly available due to the personal information (gaze data).

## References

[1] Nghi T.N., Nishijo M., Manh H.D., Tai P.T., Van Luong H., Anh T.H., Thao P.N., Trung N.V., Waseda T., Nakagawa H. (2015). Dioxins and Nonortho PCBs in Breast Milk of Vietnamese Mothers Living in the Largest Hot Spot of Dioxin Contamination. Environ. Sci. Technol..

[2] Knutsen H.K., Barregård J.A.L., Bignami M., Brüschweiler B., Ceccatelli S., Cottrill B., Dinovi M., Edler L., Grasl-Kraupp B., EFSA Panel on Contaminants in the Food Chain (CONTAM) (2018). Risk for animal and human health related to the presence of dioxins and dioxin-like PCBs in feed and food. EFSA J..

[3] Fernandez-Salguero P.M., Hilbert D.M., Rudikoff S., Ward J.M., Gonzalez F.J. (1996). Arylhydrocarbon receptor-deficient mice are resistant to 2,3,7,8-tetrachlorodibenzo-pdioxin-induced toxicity. Toxicol. Appl. Pharmacol..

[4] Mably T.A., Moore R.W., Goy R.W., Peterson R.E. (1992). In utero and lactational exposure to 2,3,7,8-tetrachlorodibenzo-p-dioxin. Toxicol. Appl. Pharmacol..

[5] Gray L.E., Kelce W.R., Monosson E., Ostby J.S., Birnbaum L.S. (1995). Exposure to TCDD during development permanently alters reproductive function in male Long Evans rats and hamsters: Reduced ejaculated and epididymal sperm numbers and sex accessory gland weights in offspring with normal androgenic status. Toxicol. Appl. Pharmacol..

[6] Gray L.E., Wolf C., Mann P., Ostby J.S. (1997). In utero exposure to low doses of 2,3,7,8-tetrachlorodibenzo-p-dioxin alters reproductive development of female Long Evans hooded rat offspring. Toxicol. Appl. Pharmacol..

[7] Takeda T., Fujii M., Hattori Y., Yamamoto M., Shimazoe T., Ishii Y., Himeno M., Yamada H. (2014). Maternal exposure to dioxin imprints sexual immaturity of the pups through fixing the status of the reduced expression of hypothalamic gonadotropinreleasing hormone. Mol. Pharmacol..

[8] Takeda T., Taura J., Hattori Y., Ishii Y., Yamada H. (2014). Dioxin-induced retardation of development through a reduction in the expression of pituitary hormones and possible involvement of an aryl hydrocarbon receptor in this defect: A comparative study using two strains of mice with different sensitivities to dioxin. Toxicol. Appl. Pharmacol..

[9] Guo Y.L., Hsu P.C., Hsu C.C., Lambert G.H. (2000). Semen quality after prenatal exposure to polychlorinated biphenyls and dibenzofurans. Lancet.

[10] Tsukimori K., Uchi H., Tokunaga S., Yasukawa F., Chiba T., Kajiwara J., Hirata T., Furue M. (2013). Blood levels of PCDDs, PCDFs, and coplanar PCBs in Yusho mothers and their descendants: Association with fetal Yusho disease. Chemosphere.

[11] Mocarelli P., Gerthoux P.M., Needham L.L., Patterson D.G., Limonta G., Falbo R., Signorini S., Bertona M., Crespi C., Sarto C. (2011). Perinatal exposure to low doses of dioxin can permanently impair human semen quality. Environ. Health Perspect..

[12] Manh H.D., Kido T., Okamoto R., Xianliang S., Viet N.H., Nakano M., Tai P.T., Maruzeni S., Nishijo M., Nakagawa H. (2013). The relationship between dioxins and salivary steroid hormones in Vietnamese primiparae. Environ. Health Prev. Med..

[13] Warner M., Rauch S., Ames J., Mocarelli P., Brambilla P., Signorini S., Eskenazi B. (2020). Prenatal dioxin exposure and thyroid hormone levels in the Seveso second generation study. Environ. Res..

[14] Knickmeyer R., Baron-Cohen S., Raggatt P., Taylor K. (2005). Fetal testosterone, social relationships, and restricted interests in children. J. Child Psychol. Psychiatry.

[15] Auyeung B., Knickmeyer R., Ashwin E., Taylor K., Hackett G., Baron-Cohen S. (2012). Effects of fetal testosterone on visuospatial ability. Arch. Sex. Behav..

[16] Auyeung B., Baron-Cohen S., Ashwin E., Knickmeyer R., Taylor K., Hackett G. (2009). Fetal testosterone and autistic traits. Br. J. Psychol..

[17] Baron-Cohen S., Auyeung B., Nørgaard-Pedersen B., Hougaard D.M., Abdallah M.W., Melgaard L., Cohen A.S., Chakrabarti B., Ruta L., Lombardo M.V. (2015). Elevated fetal steroidogenic activity in autism. Mol. Psychiatry..

[18] Silver M.K., Meeker J.D., Darbre P.D. (2021). Chapter 14—Endocrine disruption of developmental pathways and children’s health. Endocrine Disruption and Human Health.

[19] Pham N.T., Nishijo M., Pham T.T., Tran N.N., Le V.Q., Tran H.A., Phan H., Nishino Y., Nishijo H. (2019). Perinatal dioxin exposure and neurodevelopment of 2-year-old Vietnamese children in the most contaminated area from Agent Orange in Vietnam. Sci Total Environ..

[20] Pham N.T., Nishijo M., Nghiem T., Pham T.T., Tran N.N., Le V.Q., Vu T.H., Tran H.A., Phan H., Do Q. (2021). Effects of perinatal dioxin exposure on neonatal electroencephalography (EEG) activity of the quiet sleep stage in the most contaminated area from Agent Orange in Vietnam. Int. J. Hyg. Environ. Health.

[21] Van der Geest J.N., Kemner C., Verbaten M.N., van Engeland H. (2002). Gaze behavior of children with pervasive developmental disorder toward human faces: A fixation time study. J. Child Psychol. Psychiatry.

[22] Rigby S.N., Stoesz B.M., Jakobson L.S. (2016). Gaze patterns during scene processing in typical adults and adults with autism spectrum disorders. Res. Autism Spectr. Disord..

[23] Nakano T., Tanaka K., Endo Y., Yamane Y., Yamamoto T., Nakano Y., Ohta H., Kato N., Kitazawa S. (2010). Atypical gaze patterns in children and adults with autism spectrum disorders dissociated from developmental changes in gaze behaviour. Proc. Biol Sci..

[24] Thao P.N., Nishijo M., Tai P.T., Nghi T.N., Quan L.V., Anh T.H., Vu P.H.A., Nishino Y., Nishijo H. (2018). Effects of dioxin exposure on gaze behavior in 3-year-old children in Vietnam. Organohalog. Compd..

[25] Fombonne E., Rogé B., Claverie J., Courty S., Frémolle J. (1999). Microcephaly and macrocephaly in autism. J. Autism Dev. Disord..

[26] Gillberg C., de Souza L. (2002). Head circumference in autism, Asperger syndrome, and ADHD: A comparative study. Dev. Med. Child Neurol..

[27] Lainhart J.E., Bigler E.D., Bocian M., Coon H., Dinh E., Dawson G., Deutsch C.K., Dunn M., Estes A., Tager-Flusberg H. (2006). Head circumference and height in autism: A study by the Collaborative Program of Excellence in Autism. Am. J. Med. Genet. A.

[28] Crucitti J., Hyde C., Enticott P.G., Stokes M.A. (2020). Head circumference trends in autism between 0 and 100 months. Autism.

[29] The Office of the Vietnam National Steering Committee 33, Hatfield Consultants (2011). Environmental and Human Health As-Sessment of Dioxin Contamination at Bien Hoa Airbase, Viet Nam.

[30] Schecter A., Dai L.C., Papke O., Prange J., Constable J.D., Matsuda M., Thao V.D., Piskac A.L. (2001). Recent dioxin contamination from Agent Orange in residents of a southern Vietnam city. J. Occup. Environ. Med..

[31] Tawara K., Honda R., Nishijo M., Nakagawa H. (2003). Pretreatment procedure of dioxin analysis for a small volume of human breast milk. J. Kanazawa Med. Univ..

[32] Van den Berg M., Birnbaum L.S., Denison M., De Vito M., Farland W., Feeley M., Fiedler H., Hakansson H., Hanberg A., Haws L. (2006). The 2005 World Health Organization reevaluation of human and mammalian toxic equivalency factors for dioxins and dioxin-like compounds. Toxicol. Sci..

[33] Tai P.T., Nishijo M., Kido T., Nakagawa H., Maruzeni S., Naganuma R., Anh N.T., Morikawa Y., Luong H.V., Anh T.H. (2011). Dioxin concentrations in breast milk of Vietnamese nursing mothers: A survey four decades after the herbicide spraying. Environ. Sci. Technol..

[34] Pham T.N., Nishijo M., Pham T.T., Vu H.T., Tran N.N., Tran A.H., Do Q., Takiguchi T., Nishino Y., Nishijo H. (2020). Dioxin exposure and sexual dimorphism of gaze behavior in prepubertal Vietnamese children living in Da Nang, a hot spot for dioxin contamination. Sci. Total Environ..

[35] Nishijo M., Pham T.T., Nguyen A.T., Tran N.N., Nakagawa H., Hoang L.V., Tran A.H., Morikawa Y., Ho M.D., Kido T. (2014). 2,3,7,8-Tetrachlorodibenzo-p-dioxin in breast milk increases autistic traits of 3-year-old children in Vietnam. Mol. Psychiatry.

[36] Tai P.T., Nishijo M., Anh N.T., Maruzeni S., Nakagawa H., Van Luong H., Anh T.H., Honda R., Kido T., Nishijo H. (2013). Dioxin exposure in breast milk and infant neurodevelopment in Vietnam. Occup. Environ. Med..

[37] Doi H., Nishitani S., Fujisawa T.X., Nagai T., Kakeyama M., Maeda T., Shinohara K. (2013). Prenatal exposure to a polychlorinated biphenyl (PCB) congener influences fixation duration on biological motion at 4-months-old: A preliminary study. PLoS ONE.

[38] Nowack N., Wittsiepe J., Kasper-Sonnenberg M., Wilhelm M., Schölmerich A. (2015). Influence of Low-Level Prenatal Exposure to PCDD/Fs and PCBs on Empathizing, Systemizing and Autistic Traits: Results from the Duisburg Birth Cohort Study. PLoS ONE.

[39] Boraston Z., Blakemore S.J. (2007). The application of eye-tracking technology in the study of autism. J. Physiol..

[40] Chakrabarti B., Dudbridge F., Kent L., Wheelwright S., Hill-Cawthorne G., Allison C., Banerjee-Basu S., Baron-Cohen S. (2009). Genes related to sex steroids, neural growth, and social-emotional behavior are associated with autistic traits, empathy, and Asperger syndrome. Autism Res..

[41] Crider A., Thakkar R., Ahmed A.O., Pillai A. (2014). Dysregulation of estrogen receptor beta (ERβ), aromatase (CYP19A1), and ER co-activators in the middle frontal gyrus of autism spectrum disorder subjects. Mol. Autism.

[42] Dissanayake C., Bui Q.M., Huggins R., Loesch D.Z. (2006). Growth in stature and head circumference in high-functioning autism and Asperger disorder during the first 3 years of life. Dev. Psychopathol..

[43] Tai P.T., Nishijo M., Nghi T.N., Nakagawa H., Van Luong H., Anh T.H., Nishijo H. (2016). Effects of Perinatal Dioxin Exposure on Development of Children during the First 3 Years of Life. J. Pediatr..

[44] Tran N.N., Pham T.T., Ozawa K., Nishijo M., Nguyen A.T., Tran T.Q., Hoang L.V., Tran A.H., Phan V.H., Nakai A. (2016). Impacts of Perinatal Dioxin Exposure on Motor Coordination and Higher Cognitive Development in Vietnamese Preschool Children: A Five-Year Follow-Up. PLoS ONE.

[45] Pham The T., Pham Ngoc T., Hoang Van T., Nishijo M., Tran Ngoc N., Vu Thi H., Hoang Van L., Tran Hai A., Nishino Y., Nishijo H. (2020). Effects of perinatal dioxin exposure on learning abilities of 8-year-old children in Vietnam. Int. J. Hyg. Environ. Health.

[46] Pham-The T., Nishijo M., Pham-Ngoc T., Vu-Thi H., Tran-Ngoc N., Tran-Hai A., Hoang-Van L., Nishino Y., Nishijo H. (2019). Effects of prenatal dioxin exposure on children behaviors at 8 years of age of age. Proceedings of the 39th International Symposium on Halogenated Persistent Organic Pollutants—Dioxin in 2019.

[47] Vu H.T., Nishijo M., Pham T.N., Pham-The T., Hoanh L.V., Tran A.H., Tran N.N., Nishino Y., Do Q., Nishijo H. (2021). Effects of perinatal dioxin exposure on mirror neuron activity in 9-year-old children living in a hot spot of dioxin contamination in Vietnam. Neuropsychologia.

[48] Ames J., Warner M., Siracusa C., Signorini S., Brambilla P., Mocarelli P., Eskenazi B. (2019). Prenatal dioxin exposure and neuropsychological functioning in the Seveso Second Generation Health Study. Int. J. Hyg. Environ. Health.

[49] Vreugdenhil H.J., Slijper F.M., Mulder P.G., Weisglas-Kuperus N. (2002). Effects of perinatal exposure to PCBs and dioxins on play behavior in Dutch children at school age. Environ. Health Perspect..

[50] Winneke G., Ranft U., Wittsiepe J., Kasper-Sonnenberg M., Fürst P., Krämer U., Seitner G., Wilhelm M. (2014). Behavioral sexual dimorphism in school-age children and early developmental exposure to dioxins and PCBs: A follow-up study of the Duisburg Cohort. Environ. Health Perspect..

[51] Poland A., Knutson J.C. (1982). 2,3,7,8-tetrachlorodibenzo-p-dioxin and related halogenated aromatic hydrocarbons: Examination of the mechanism of toxicity. Annu. Rev. Pharmacol. Toxicol..

[52] Peterson R.E., Theobald H.M., Kimmel G.L. (1993). Developmental and reproductive toxicity of dioxins and related compounds: Cross-species comparisons. Crit. Rev. Toxicol..

[53] Takeda T., Matsumoto Y., Koga T., Mutoh J., Nishimura Y., Shimazoe T., Ishii Y., Ishida T., Yamada H. (2009). Maternal exposure to dioxin disrupts gonadotropin production in fetal rats and imprints defects in sexual behavior. J. Pharmacol. Exp. Ther..

[54] Hattori Y., Takeda T., Nakamura A., Nishida K., Shioji Y., Fukumitsu H., Yamada H., Ishii Y. (2018). The aryl hydrocarbon receptor is indispensable for dioxin-induced defects in sexually-dimorphic behaviors due to the reduction in fetal steroidogenesis of the pituitary-gonadal axis in rats. Biochem. Pharmacol..

[55] Furue M., Ishii Y., Tsukimori K., Tsuji G. (2021). Aryl Hydrocarbon receptor and dioxin-related health hazards-lessons from Yusho. Int. J. Mol. Sci..

[56] Dobs K., Bülthoff I., Schultz J. (2018). Use and Usefulness of Dynamic Face Stimuli for Face Perception Studies-a Review of Behavioral Findings and Methodology. Front. Psychol..

